# The Lasker~Koshland Special Achievement Award in Medical Science awarded to Lucy Shapiro

**DOI:** 10.1073/pnas.2519777122

**Published:** 2025-09-11

**Authors:** Leslie B. Vosshall

**Affiliations:** ^a^HHMI, Chevy Chase, MD 20815; ^b^Laboratory of Neurogenetics and Behavior, The Rockefeller University, New York, NY 10065; ^c^Kavli Neural Systems Institute, The Rockefeller University, New York, NY 10065

## Abstract

Scientists can contribute to society in numerous ways. Some scientists discover new biological principles and found entirely new fields. Some scientists are inspiring mentors and create the next generation of inclusive lab leaders. Some scientists are entrepreneurs who develop clinically effective therapeutics. Some scientists are trusted advisors to government and pharmaceutical companies. Some scientists are visionary institutional leaders who build new departments. From this menu of activities, most scientists select two or at most three. It is exceedingly rare for a single scientist to excel in all of these areas, consistently, over the course of a career. Lucy Shapiro is this extraordinary scientist. She founded the field of bacterial cell biology and trained the next generation of microbiologists, launched biotech companies to develop new antifungal drugs, served as an unofficial advisor to two presidential administrations and numerous companies, institutions, and foundations, and built and led successful academic departments. The 2025 Lasker~Koshland Special Achievement Award in Medical Science is awarded to Lucy Shapiro “for a 55-y career in biomedical science—honored for discovering how bacteria coordinate their genetic logic in time and space to generate distinct daughter cells; for founding Stanford’s distinguished Department of Developmental Biology; and for exemplary leadership at the national level.”

## A Scientist Grows in Brooklyn

Born in Brooklyn in 1940 as the eldest of three daughters of Yetta and Philip Cohen, Shapiro grew up in an immigrant household of modest means where education was highly prized (1). From a young age, she was tasked with taking care of her younger sister Enid, who had special needs. Both parents worked and there was no possibility of hiring a nanny. Shapiro bore this serious responsibility with the kind of will-do spirit that has swept away all obstacles in her path in the decades since.

As high school approached, her parents were concerned that the local Manual Training High School, that was decidedly not a college preparatory institution with only 3% of graduates pursuing education beyond high school (1), was not up to the task of educating their brilliant daughter. Instead, it was decided she would audition for the High School of Music and Art (now the Fiorello H. LaGuardia High School of Music & Art and Performing Arts) in the music track given that she had been taking piano lessons since she was 4 y old. But Shapiro had a different plan. At thirteen, with characteristic determination, she embarked on a secret mission. She borrowed Jon Gnagy’s classic “Learn to Draw” from the local public library, then every night after her family went to bed, she taught herself to draw. When she prepared her application, she secretly checked the “art” rather than the “music” box. Her self-taught art portfolio gained her admission to this legendary Manhattan high school and with it the lesson that she had the power to decide the direction of her life.

When it came time for college, Shapiro was desperate to get out of Brooklyn but there was no money, and so she enrolled in an experimental honors program at Brooklyn College, which offered a world class and at the time a free college education (1). At Brooklyn College, Shapiro double majored in biology and fine arts with a focus on the Italian Renaissance and worked as a medical illustrator to support herself. At a group art show displaying her paintings, she met the physical chemist Theodore Shedlovsky, a professor at what is now The Rockefeller University who was a self-appointed talent scout for artistic young people who he believed should be scientists (1, 2). Recognizing something special in Shapiro, Shedlovsky convinced her to take an organic chemistry course. This was a transformative moment in Shapiro’s life and inspired her to pivot from a path of art to a path of science. She pursued graduate work with Jerry Hurwitz and Tom August originally at New York University and moved with their lab to the Albert Einstein College of Medicine where she earned her PhD in 1966.

Because Shapiro had no formal education in math or science, Hurwitz sent her to institutions around New York City to take classes in theoretical organic chemistry, statistical mechanics, and mathematics (1). Rather than studying at the Einstein campus in the Bronx, Shapiro retreated to the peaceful interior courtyard of the Frick Museum on Fifth Avenue in Manhattan. There to the burbling sound of the fountain and surrounded by an extraordinary art collection, Shapiro immersed herself in new knowledge, surely the only museum visitor with a slide rule and thick physics textbooks.

**Fig. 1.**
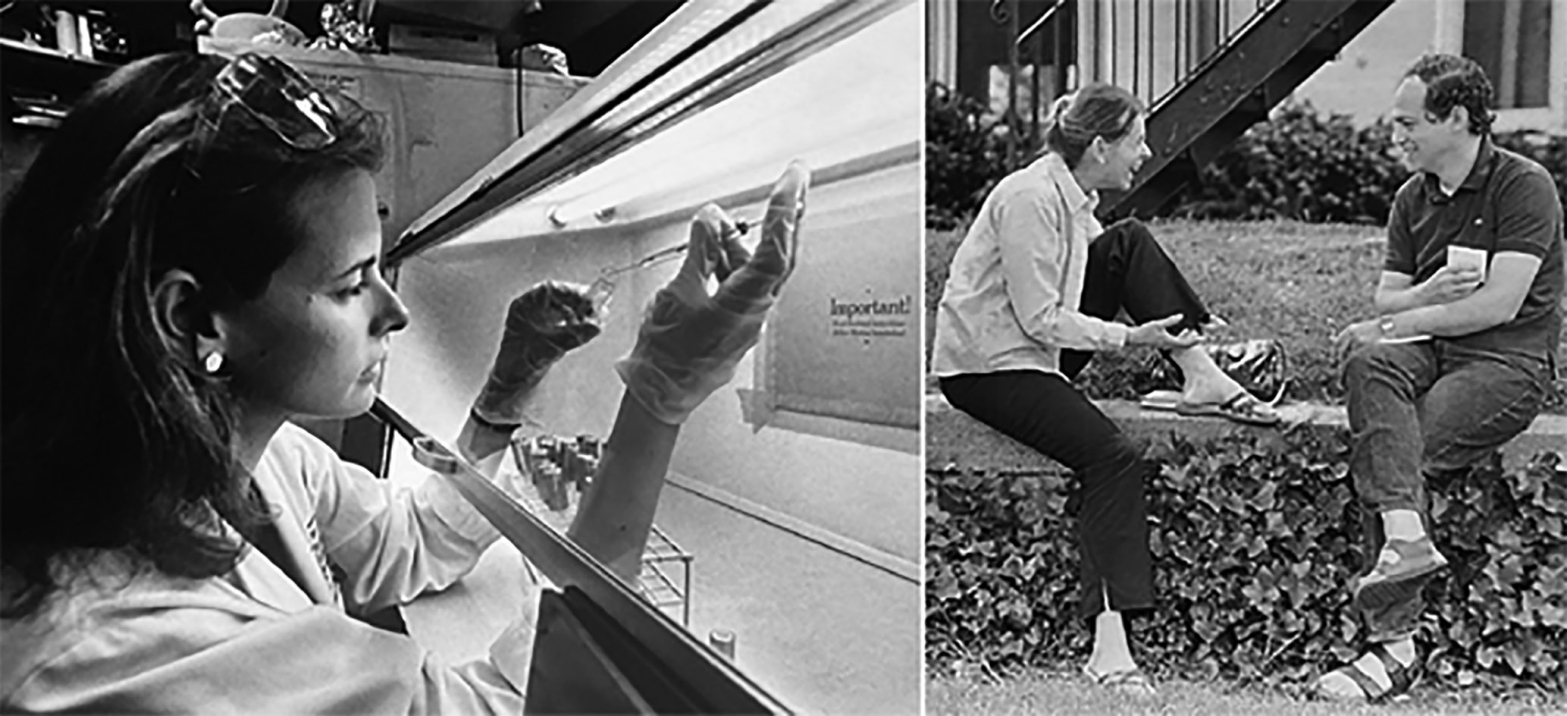
Lucy Shapiro in her lab at Albert Einstein College of Medicine approximately 1980 (*Left*). Lucy Shapiro and Richard Losick at Cold Spring Harbor Laboratory in 1985. Image credit: Lucy Shapiro (Stanford University, Stanford, CA).

Recognizing her huge potential, her mentors sent her to the Cold Spring Harbor Laboratory for a crash course in biology by enrolling her in two consecutive summer courses: bacterial genetics and Max Delbrück’s influential bacteriophage course (3). Soon, Shapiro was fully embedded in the Cold Spring Harbor community of molecular biology, spending several summers there (Fig. 1). This was at the dawn of molecular biology history and Shapiro was right in the middle of it, with the Nobel prize-winning geneticist Barbara McClintock becoming an important mentor. This was the beginning of a meteoric rise of a young superstar geneticist and molecular biologist.

## A Bold and Original Vision and Founding the Field of Bacterial Cell Biology

Shortly after Shapiro received her PhD in molecular biology in 1966, Bernard Horecker, chair of the molecular biology department at Albert Einstein College of Medicine, asked her to return as a faculty member with an extraordinary offer to pick any scientific question of her choice. “He told me to take three months to read and think. That does not happen today! It was a gift.” (4). During three months of intensive reading and reflection, she identified critical limitations in contemporary approaches to biological research. Scientists were either conducting reductionist biochemical studies on isolated cellular components or performing genetic experiments to infer cellular mechanisms. Neither approach satisfied her curiosity about how cells actually function as integrated, three-dimensional systems. To frame her choice of a research program for her new lab, Shapiro posed two fundamental questions that continue to drive biological research: How do living organisms translate information from a linear genetic code into three-dimensional structures? And how do the many biochemical processes that scientists study in isolation actually interconnect within intact living cells?

Shapiro’s search for an appropriate experimental system to address these two questions led her to *Caulobacter crescentus*, an obscure bacterium that would become central to a revolution in cell biology. This organism possessed exactly the characteristics she needed: it divides asymmetrically, producing two morphologically distinct daughter cells that could be easily distinguished under a light microscope. When *C. crescentus* divides, one daughter cell—the swarmer—carries a flagellum at one pole, enabling it to swim to new locations. The other daughter carries a stalk at one pole, which tethers it to surfaces. After division, the stalked cell immediately begins a new cell cycle, while the swarmer’s division activities remain blocked until it propels itself to a new location and differentiates into a stalked cell. This asymmetric division pattern provided Shapiro with a unique opportunity to map molecules and structures relative to distinct, visible appendages throughout the cell cycle. The system allowed her to investigate how cells allocate their contents during division, ensuring that each daughter receives appropriate components for its designated fate.

Armed with her organism of choice, Shapiro led a major revolution in how scientists viewed bacterial cells. The prevailing wisdom depicted bacteria as simple organisms where proteins dispersed evenly throughout the cell, creating what Shapiro has described as “swimming pools” where proteins float everywhere in an unorganized manner. Shapiro discovered that proteins called chemoreceptors localize specifically at cell poles in both *C. crescentus* (5) and the traditional model bacterium *Escherichia coli* (6). Since *E. coli* produces identical daughter cells, these observations established that protein sequestration to particular cellular sites represented a fundamental organizing principle of bacteria in general rather than an oddity of *Caulobacter* biology. This discovery fundamentally challenged the concept of bacteria as a collection of jumbled macromolecules contained within a cell wall. Instead, Shapiro demonstrated that microbes place their components in specific locations at specific times, revealing an unexpected level of cellular organization and sophistication for a single-celled prokaryotic organism.

Shapiro’s investigation of temporal control mechanisms revealed how molecular machinery drives cell cycle progression. She discovered that specific cellular equipment performs required tasks in controlled fashion—DNA replication occurs once per cycle, and cell constriction machinery operates only after genetic material has been properly distributed to each daughter cell. By the late 1990s, genome-wide analysis techniques allowed Shapiro to reveal the genetic foundations of this process. She demonstrated that a large subset of *C. crescentus* genes turn on and off at specific times during the cell cycle, indicating that sophisticated temporal regulation rather than random gene expression patterns underlie cell division and differentiation.

The research identified three master regulatory proteins—DnaA, GcrA, and CtrA—that orchestrate cell cycle progression by acting consecutively to control numerous downstream genes (7). This regulatory cascade operates through an elegant mechanism involving DNA methylation states that couple replication progression to gene expression timing. One of Shapiro’s most significant discoveries involved the relationship between DNA replication and gene regulation through methylation patterns (8). At the cell cycle’s beginning, both DNA strands carry methyl groups. However, newly synthesized DNA initially lacks these chemical modifications, creating hemimethylated DNA. The methylation state of any chromosomal region depends on how recently the replication machinery has passed through it. This creates a “methylation ratchet” where sequential changes in chromosomal methylation states couple DNA replication progression to cell cycle events controlled by the master regulatory proteins. This mechanism ensures that dnaA and ctrA genes—encoding the DnaA and CtrA regulatory proteins—rise and fall in activity as they are duplicated, creating precise temporal control over cell cycle progression. The system displays remarkable sophistication: CtrA suppresses DNA replication until appropriate timing while DnaA enables replication initiation. CtrA is an extraordinary example of a single protein that is both a master transcription factor that regulates morphological features like the bacterial flagellum and pili and coordinates a web of cell division cycle genes, and that is also a protein that binds to the bacterial origin of replication and blocks initiation of DNA synthesis (9).

Shapiro’s spatial studies extended beyond protein localization to encompass the organization of entire chromosomes. By the turn of the 21st century, scientists knew that genes resided at particular chromosomal addresses, but genetic material was thought to drift freely within bacterial cells. Using time-lapse microscopy and fluorescent tagging, Shapiro demonstrated in 2004 that each chromosomal region moves in an orderly fashion to predetermined locations as it undergoes duplication. DNA replicated early in the cycle positions itself near the stalked pole, while later-replicated regions locate near the division plane (10). This work established that bacterial genomes exhibit much higher spatial organization than previously imagined. The research revealed detailed mechanisms facilitating chromosomal movement. The first-replicated portion travels rapidly to the opposite pole, and subsequent orchestrated events ensure that cell division apparatus formation waits until chromosomes have properly segregated to daughter cells.

Shapiro’s research revealed that Caulobacter’s asymmetric division represents an evolutionary strategy predating multicellularity. The parent cell makes a nonaltruistic decision to produce two distinct daughters: a swarmer that moves to new positions and a stalked cell that determines whether the current location remains viable. This strategy enhances species fitness and survival probability by diversifying environmental sampling. The process involves critical regulatory mechanisms including two-component signaling, cyclic dinucleotides, methylation, phosphorylation, and regulated proteolysis (11–13). These mechanisms work together to ensure proper cell fate determination and to coordinate complex developmental programs within single cells.

Since 1995, Shapiro has conducted much of her research with physicist Harley McAdams. Their collaboration began when Shapiro’s enthusiasm about *Caulobacter* biology convinced McAdams that strong analogies exist between biological and electrical control systems. Their interdisciplinary approach placed physicists next to bacterial geneticists and electrical engineers alongside biochemists in the same lab. This cross-pollination generated novel insights, including the recognition that genetic and electrical circuitry share fundamental features. The cell cycle control network operates as an integrated system maintaining robust function under fluctuating internal conditions and environmental perturbations (14). This work helped establish systems biology as a distinct field, demonstrating that understanding biological networks requires knowledge not only of key molecules and their functions but also their precise locations in time and space within developing cells.

**Fig. 2.**
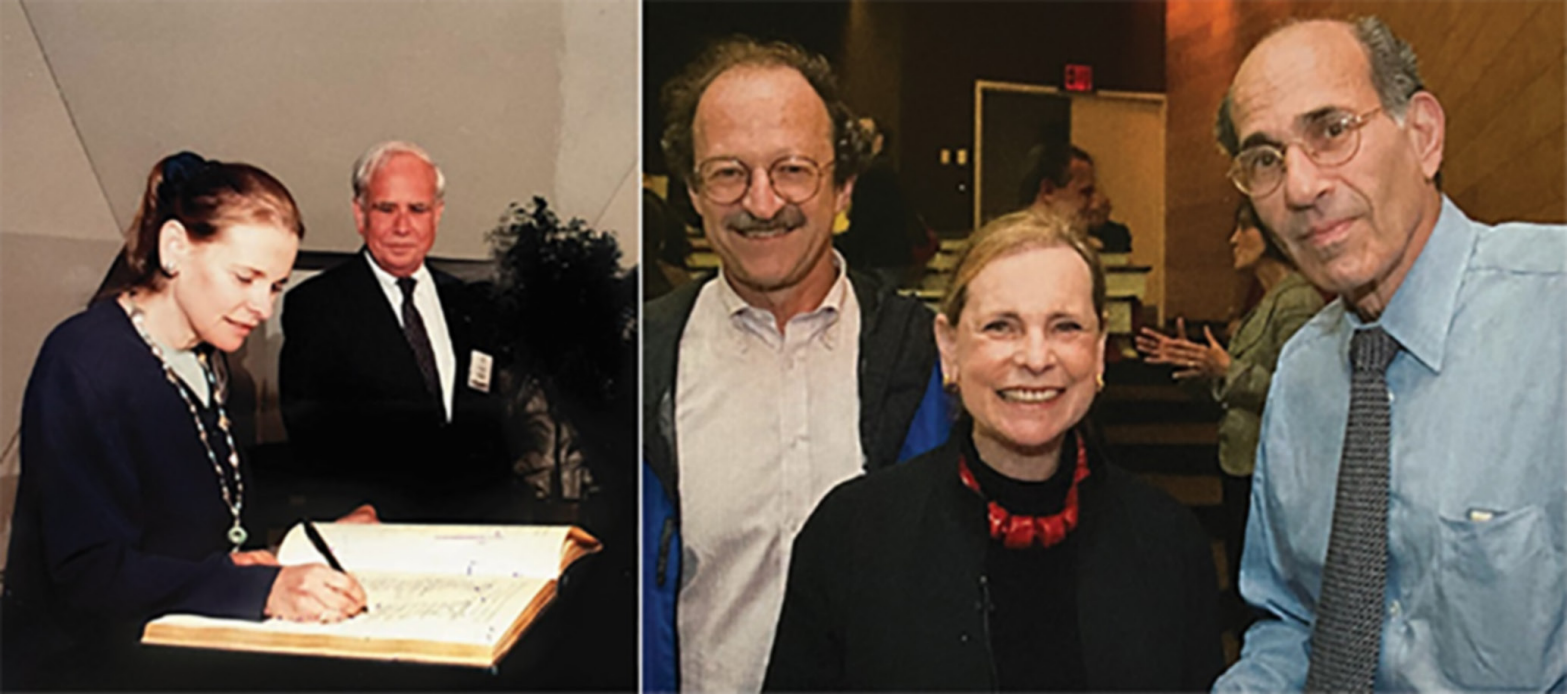
Lucy Shapiro signing the Registry of Membership at her induction into the National Academy of Sciences in 1994 while Peter H Raven, Home Secretary, looks on (*Left*). Harold Varmus, Lucy Shapiro, and Richard Axel at Columbia University in New York in 1994 (*Right*). Image credit: Lucy Shapiro (Stanford University, Stanford, CA).

Taken together, Lucy Shapiro’s pioneering research with *C. crescentus* has revealed universal principles governing cellular organization, gene regulation, and developmental biology that extend across all life forms. Shapiro’s discoveries resonate far beyond bacterial biology. The asymmetric division process she elucidated shares striking similarities with stem cell division, where two distinct cells arise—one identical to the parent and one differentiated. Her work provides detailed understanding of how a single genome produces different cellular outcomes, a phenomenon underlying all multicellular life. For her transformative scientific discoveries, Shapiro has been honored with election to the National Academy of Sciences (Fig. 2), the Canada Gairdner International Award in 2009, and the 2011 US National Medal of Science presented by President Obama, among many other honors.

Beyond impressive publications and prestigious prizes, Shapiro has shown that it is not just doing great science and building fields but also establishing a legacy of scientific progeny to carry on the work that matters. Accordingly, her mentoring record is outstanding. She has trained 71 graduate students and postdoctoral scientists, and more than 30 are now running their own labs. Remarkably, twenty-four former Shapiro Lab mentees are now running independent *Caulobacter* laboratories in the United States, Canada, and Europe, including two who were selected as Howard Hughes Medical Institute Investigators. This is remarkable because Shapiro’s administrative and service responsibilities were a significant draw on her time, and yet she managed to keep her lab open and thriving over decades.

## From a Bold Idea to New Antifungal Drugs

Shapiro’s contributions did not end with fundamental biological discoveries. Teaming up with Penn State chemist Steve Benkovic, Shapiro embarked on an audacious quest to develop novel antifungal therapeutics based on the idea of replacing carbon atoms with boron, an approach that pharmaceutical chemists assured the duo would never work. Their breakthrough came from Shapiro’s discovery of a unique DNA methylation mechanism in alpha bacteria like Caulobacter, where an essential adenine DNA methyltransferase operates within a narrow window following chromosome replication, creating a biological clock that coordinates the cell cycle (8, 15). Recognizing this enzyme as an ideal therapeutic target, Benkovic synthesized novel boron-containing compounds that not only inhibited this specific mechanism but proved effective against gram-negative bacteria in general (16). This pioneering work led to the founding of Anacor Pharmaceuticals, which developed two FDA-approved drugs: tavaborole (Kerydin) for nail fungus and crisaborole (Eucrisa) for atopic dermatitis—marking one of only two new antifungal agents developed in 25 y. The incorporation of boron into pharmacophores had been virtually unexploited territory, and their success opened an entirely new frontier in drug development. Following Anacor’s acquisition by Pfizer, Shapiro and Benkovic turned their attention to global agricultural challenges, founding Boragen (now 5Metis) to combat devastating crop diseases in developing countries. Their boron-based compounds proved effective against Black Sigatoka, the fungal blight caused by *Mycosphaerella fijiensis* that has evolved resistance to several fungicides and is threatening banana crops worldwide, as well as pathogens affecting sorghum and other vital food sources.

## Born to Lead

Lucy Shapiro’s academic leadership trajectory demonstrates a consistent pattern of institutional transformation and visionary department building. Just 10 y after joining Einstein as an assistant professor, she was appointed to chair that department and 4 y later the entire Division of Biological Sciences. In 1986, Columbia University Medical Center recruited her to chair their Department of Microbiology and Immunology. After only 1 y at Columbia, Stanford contacted Shapiro to try to recruit her away from New York, an offer she immediately refused – she had just moved to Columbia and was not interested. Two further years of pestering by Stanford with consistent escalation in the quality of the offer still did not yield a “yes” from Shapiro. This changed one day in 1989 when Dave Hogness arrived unannounced in her office carrying a dozen roses and blueprints for the new Beckman Center and an offer she could not refuse: the opportunity to serve as Inaugural Chair of their newly conceived Department of Developmental Biology (1). This role allowed Shapiro to build something entirely new, personally recruiting faculty and establishing the departmental culture and goals. Her approach to talent development proved remarkably successful. Today, the Stanford Department of Developmental Biology is internationally recognized. Nine of its 28 tenured faculty became Howard Hughes Medical Institute investigators and fourteen were elected to the National Academy of Sciences. Shapiro’s influence at Stanford extended well beyond her own department. She became an early advocate for Bio-X, the interdisciplinary bioscience institute that has significantly impacted Stanford’s scientific enterprise by fostering collaboration across traditional disciplinary boundaries. Her commitment to integrated research approaches led to her appointment as Director of the Beckman Center for Molecular and Genetic Medicine. The Beckman Center represents the culmination of her leadership philosophy, housing three basic science departments—Biochemistry, Molecular & Cellular Physiology, and Developmental Biology—along with dozens of laboratories and core facilities. Under her direction, the Center has thrived as a hub that promotes scientific integration not only across departments but also from basic research to translational applications. Each leadership position in Shapiro’s career built upon the previous one, creating a legacy of institutional excellence and collaborative scientific culture that continues to influence research and education. Her ability to identify talent, foster interdisciplinary collaboration, and build lasting institutional frameworks has left an indelible mark on each organization she has led.

## Trusted Advisor to Government and Pharmaceutical and Academic Sectors

In the realm of professional service, Shapiro’s contributions are impressive in their intensity, breadth, and altruism. Shapiro appears to be everywhere advising everyone. A colleague recently quipped “I can’t remember a time when I wasn’t serving on a review board with Lucy,” and indeed she has served on a wide variety of government, institutional, nonprofit, and corporate boards in the United States, Europe, and Asia. She served on the Board of Directors of SmithKline Beecham and later GlaxoSmithKline after their merger, plus Pacific Biosciences, Silicon Graphics, and Gen-Probe. Much of her work with private foundations has involved evaluating candidates for early career fellowships, yet another way that Shapiro supports the next generation of scientists.

Her high-level government advisory roles began when President Bill Clinton invited a small group of microbiologists including Joshua Lederberg, Craig Venter, and Shapiro to attend a Cabinet Meeting in 1998 to discuss genetic engineering and biological weapons. Shapiro deviated from her brief scripted remarks on bioterrorism to give Clinton an extended lesson in the genetics of antibiotic resistance and the importance of understanding how nature can generate pathogens. This led her to become an informal advisor to staffers in both the Clinton and George W. Bush administrations, including sitting in the White House Situation Room unofficially advising Homeland Security Secretary Tom Ridge and Secretary of State Condoleezza Rice specifically on bioterrorism threats (1). Presciently, just before the COVID-19 pandemic, Shapiro addressed the Senate Armed Services Committee to warn the government about future pandemics.

## Legacy of Special Achievement

In conclusion, Lucy Shapiro’s special achievements are truly special: she is a creative innovator in science, an outstanding mentor, an entrepreneur, and a charismatic leader in science. Over five decades and nearly 350 papers, Shapiro created an entirely new field. She transformed *C. crescentus* from a little-known bacterium into one of the most powerful experimental systems for understanding bacterial cell cycle control and cell fate determination. Her work fundamentally changed our understanding of bacterial cells from simple bags of enzymes to sophisticated, spatially organized systems with integrated genetic circuits.

Her pioneering contributions launched two influential areas of biological sciences: bacterial cell biology (6) and systems biology (14). By demonstrating that complete explanations of cellular networks require understanding not only what regulatory molecules do but also where they are in time and space, Shapiro established paradigms that continue to guide research across multiple biological disciplines. Her approach of combining interdisciplinary methods—genetics, biochemistry, advanced imaging, and computational modeling—has become the standard for understanding complex biological systems as integrated wholes.

Lucy Shapiro has been a transformational presence in science. At a time when few women were scientists or held leadership positions, her brilliance and determination and will-do spirit demonstrated that women could compete at the highest levels. Her scientific acumen and confidence and charisma propelled her to success not only in research but also in leading departments, founding biotech companies, and advising the US government on antibiotic resistance, bioterrorism, and pandemics. By normalizing women’s presence in these male-dominated spheres, she leveled the playing field for every woman who followed. As best said by Richard Axel (Fig. 2), “Lucy provides the best evidence that one can be brilliant and deeply human.”

## Data Availability

There are no data underlying this work.
